# Low MXene Loading of Epoxy Composite with Enhanced Hydrothermal Resistance

**DOI:** 10.3390/polym17091229

**Published:** 2025-04-30

**Authors:** Mengke Jing, Shujie Zhang, Sichang Zhang, Mingzhou Li, Fan Chen, Yuchen Ma, Bo Sun

**Affiliations:** 1 School of Textile Science and Engineering, Tiangong University, Tianjin 300387, China; 2 Beijing Gas Huanneng Engineering & Technologies Co., Ltd., Beijing 100020, China

**Keywords:** epoxy resin, MXene, nanocomposites, hydrothermal aging

## Abstract

This work focuses on the hydrothermal aging of two-dimensional layered Ti_3_C_2_T_x_ (MXene)/epoxy (EP) nanocomposites. MXene/EP composites were successfully prepared by homogeneously dispersing multilayer MXene (m-MXene) and few-layer MXene (f-MXene) into the curing agent, methyl nadic anhydride (MNA). Considering the application, the MXene loading was designed to be 0.1 wt.%. Characterization included the characteristics of MXene, the water absorption behavior of the resin and composite samples, the glass transition temperatures (*T*_g_) in various states, and the tensile strength evolution during aging. The curing behavior of the MXene composites was also discussed to facilitate an understanding of the processability. The results showed that MNA can chemically bond with MXene to obtain a stable suspension. The addition of MXene increased the curing characteristic temperature of the system, but the change in the activation energy of the curing reaction was minimal. The addition of MXene decreased the crosslink density of the epoxy resin, leading to a decrease in the *T*_g_ value of the initial samples. After hydrothermal aging, the *T*_g_ of pure EP decreased by 46.9 °C, and re-drying the samples did not fully restore the *T*_g_. However, the *T*_g_ of the MXene/EP system decreased by only 8.9 °C (m-MXene) and 9.5 °C (f-MXene), respectively, and the *T*_g_ values of the samples were fully restored to their pre-aging levels via re-drying. Experiments with immersion at 25 °C and 100 °C showed that the difference in water absorption behavior between the MXene/EP and pure EP systems was minimal. Tensile tests showed that the addition of MXene increased the initial strength of the resin system by 14.7% (m-MXene) and 20.9% (f-MXene). After 400 h of hydrothermal aging, the tensile strength retention of the pure EP samples was 69.1%, while the strength retention of the MXene/EP samples was 85.3% (m-MXene) and 83.0% (f-MXene). The combined results demonstrate that the addition of MXene with a low loading of only 0.1% can effectively improve the hydrothermal resistance of epoxy resins.

## 1. Introduction

Epoxy resins are a class of thermosetting polymers that exhibit numerous advantageous properties, including excellent mechanical strength, corrosion resistance, electrical insulation, low shrinkage, and high adhesion [1,2,3]. These polymers find wide application in a variety of industrial fields, including the manufacture of adhesives, anticorrosive coatings, matrices for composites, and insulators for electronic components. Water often has an adverse effect on the properties of cured EP, particularly under high-temperature conditions. The aging issue in hydrothermal or hygrothermal environments is a major focus of epoxy resin research.

Due to the macromolecular network structure of thermosetting polymers, water molecules can typically permeate [4]. The absorbed moisture can be classified into free water and bound water [5]. Water molecules that diffuse into the free volume of the resin usually do not cause swelling and plasticization, whereas bound water, which is attracted to the polar sites within the molecular structure, is associated with swelling and plasticization [6]. Water absorption can induce reversible and irreversible changes in the composition and properties of materials. Some aging effects, such as softening and plasticization, are reversible during the initial stages of hydrothermal aging. However, prolonged environmental exposure can result in permanent and irreversible changes to the properties [7]. The possible mechanisms for the degradation of resin properties during hydrothermal or hygrothermal processes include swelling and plasticization, microcracking, degradation of the molecular chain network, and polymer relaxation [7,8,9]. For fiber-reinforced composites, water also weakens the bond between the fibers and the matrix [10]. In addition, physical aging due to high temperatures is also an important mechanism for the deterioration of the epoxy resin properties. Physical aging involves the simultaneous reduction in the free volume and conformational changes of the crosslinked molecular structure when exposed to sub-*T*_g_ temperatures for extended periods of time [11]. The effects of the above aging effects on the properties of epoxy resins have been widely observed in many experiments. Recently, the effect of water absorption on the mechanical behavior of resins has begun to receive attention in the field of numerical research. For example, the damage model of Rocha et al. considers the changes in the behavior of polymers after moisture absorption [12].

The addition of nanofillers is a promising method for improving the hydrothermal resistance of epoxy resins. Many scholars have studied the use of graphene, carbon nanotubes, and other materials to improve the hydrothermal or hygrothermal resistance of epoxy resins. Starkova et al. [13] investigated the thermal-mechanical properties of epoxy nanocomposites filled with thermally reduced graphene oxide and multi-walled carbon nanotubes after exposure to hot distilled water. They found that adding low contents (0.3 wt.%) of nanofillers significantly reduced the water absorption capacity of the epoxy polymer, limited the decrease in glassy and rubbery moduli, limited the decrease in the glass transition temperature, and improved its resistance to hydrothermal aging. The study also found that the addition of graphene oxide was more effective than carbon nanotubes. In another study, Starkova et al. [14] incorporated amino-functionalized graphene oxide nanoparticles into epoxy resin (up to 1.72 wt.%). The nanocomposites were characterized by a lower water diffusivity and decreased leaching effects. The glassy and rubbery storage moduli and *T*_g_ increased, and the degradation effect of hydrothermal aging on the properties of the nanocomposites was significantly reduced. Glaskova-Kuzmina et al. [15] investigated the hydrothermal effect on the flexural and thermomechanical properties of epoxy resins containing multi-walled carbon nanotubes and carbon nanofibers as well as their hybrid components. They established a property prediction model for predicting the elastic modulus of specimens subjected to both water absorption and thermal aging. The study found that the addition of carbon nanofillers improved the hydrothermal aging properties of the epoxy resins, including their adsorption, flexural, and thermomechanical characteristics. It was also found that most composites filled with hybrid carbon nanofillers exhibited the best performance. In another work by Glaskova-Kuzmina et al. [16], the reversibility of water on the thermomechanical properties was investigated. The study focused on the hygroscopic–desorption–reabsorption characteristics of multi-walled carbon nanotube/epoxy-based nanocomposites. They found that water absorption was not completely reversible. Nanofillers limited the irreversible damage to the thermomechanical properties from aging. The addition of two types of multi-walled carbon nanotubes in epoxy resin reduced the adsorption characteristics of all the adsorption tests and decreased the swelling rate after hygroscopic desorption. Prolongo et al. [17] studied the hydrothermal resistance of epoxy resins reinforced with graphene nanoplatelets with different geometries and aspect ratios (thickness and lateral dimensions). They found that the addition of graphene nanoplatelets increased the glass transition temperature and stiffness and enhanced the barrier properties of the epoxy matrix. The effectiveness of the nanoplatelets increased with their specific surface area while their dispersion degree was suitable. Thinner nanoplatelets tended to wrinkle, decreasing their efficiency as nanofillers.

Transition metal carbides and nitrides (MXenes) are a class of emerging two-dimensional (2D) materials that have attracted significant attention since the discovery of the first MXene (Ti_3_C_2_T_x_) [18]. MXenes possess a typical layered structure and are derived from hexagonal-layered ternary-transition-metal carbides and nitrides (MAX phases, explained as follows). M represents an early transition metal, A is an element from group 13 or 14 (such as Al, Zn, Si, etc.), and X indicates C or N [19]. Element A is etched during the synthesis of MXene. Functional groups “T” are decorated on the surface of MXenes, such as -O, -OH, and -F, and are ultimately described by the formula M_n+1_X_n_T_x_ (*n* = 1, 2, 3) [19]. MXenes exhibit numerous outstanding properties, including high strength and elastic modulus, high chemical stability, and excellent conductivity [20]. These exceptional characteristics make them suitable for applications in energy conversion and storage, sensors, optoelectronic devices, electromagnetic and environmental applications, and as anticorrosion materials [21,22,23]. In recent years, MXenes have also been used to prepare nanocomposites to enhance the performance of polymers, such as mechanical properties, electromagnetic shielding, and corrosion resistance. These reviews [21,24] help to understand the application of MXene loading in epoxy resins. However, research on the hydrothermal aging of MXene/epoxy composites is lacking. Carey et al. [25] successfully prepared well-dispersed 5.0 wt.% Ti_3_C_2_T_x_ MXene epoxy resin nanocomposites and investigated their water transport and mechanical properties. The study found that the addition of MXene slightly improved the mechanical properties and thermal stability of the epoxy resin. The samples were subjected to steam adsorption experiments under various temperature and humidity conditions. The results showed that the water absorption and diffusion rate of the MXene/epoxy nanocomposites were lower than that of the pure resin. Lu Liu et al. [26] functionalized Ti_3_C_2_T_x_ and prepared MXene/epoxy resin composites with uniform dispersion. They investigated the effect of MXene on the mechanical and physical bonding of the epoxy resin. The results showed that the addition of 0.2 wt.% MXene increased the tensile strength and flexural strength by 51% and 32%, respectively. The glass transition temperature decreased due to the reduced crosslink density of the epoxy matrix. In addition, MXene was found to improve the thermal conductivity and electrical conductivity of the epoxy resin composites. Other studies [27,28,29,30,31] have investigated the curing behavior, mechanical properties, and thermal properties of MXene/EP nanocomposites. Numerous researchers [32,33,34] have also studied MXene/EP coatings as metal anticorrosion coatings. These studies found that MXenes enhance the barrier properties of the coatings, preventing the diffusion of corrosive agents into the substrate. However, these studies did not discuss the role of MXene in the hydrothermal aging of epoxy resin composites. To the best of our knowledge, no research has yet focused on the hydrothermal aging of MXene/EP composites. As a material with a physical structure similar to graphene, using MXene to enhance the hydrothermal aging resistance of epoxy resins should have considerable potential.

Our work focuses on the hydrothermal aging of MXene/EP composites. Multilayer and single-layer Ti_3_C_2_T_x_ MXenes were successfully uniformly dispersed in the hardener MNA, and covalently bonded MXene/EP composites were prepared. Considering the high production cost of MXene, for the feasibility of application, the load of MXene was only 0.1 wt.%. By characterizing the water absorption behavior of the resin samples and composite samples, the glass transition temperature under various conditions, and the evolution of tensile strength during aging, the role of MXene in enhancing the hydrothermal aging resistance of epoxy resins was explored. In addition, the curing behavior of MXene composites is also discussed to understand the processability of curing. This study helps to expand the application field of MXene and provides a new perspective for the development of hydrothermal resistant epoxy resin composites.

## 2. Experiments

**Figure 1:**
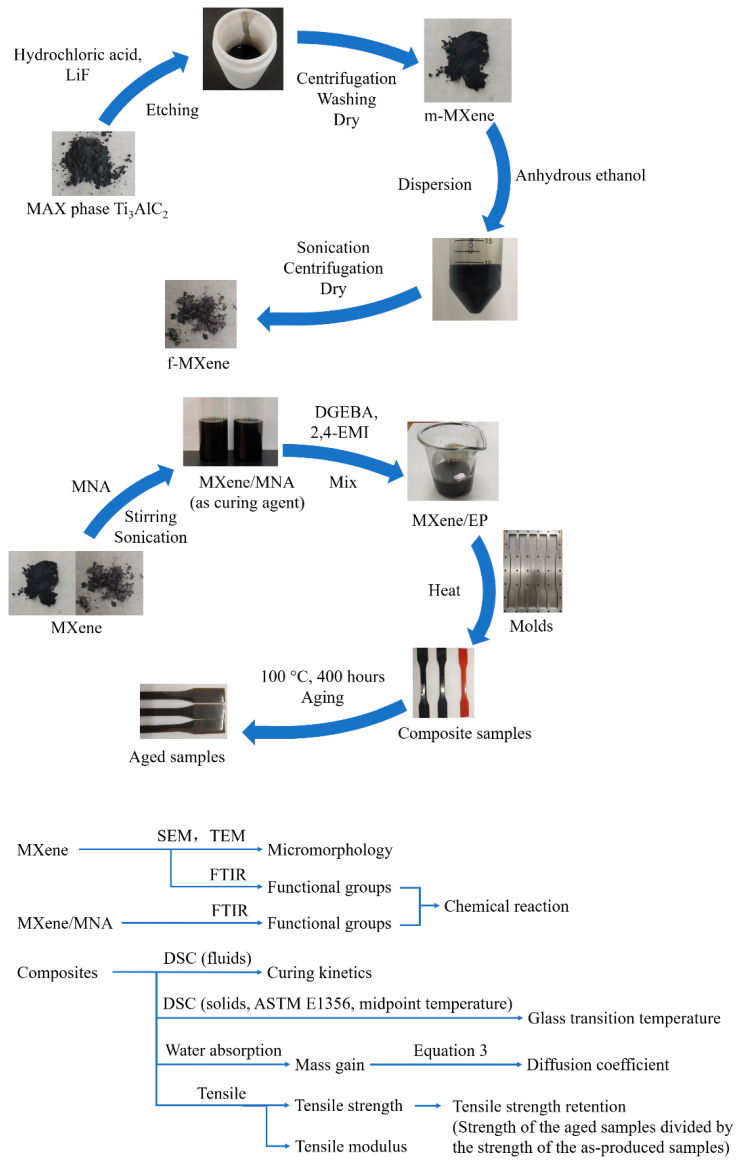
Workflow diagram of the experimental process.

Figure 1 illustrates the workflow of the experimental part. The following sections describe more detailed information.

### 2.1. Materials

MAX phase Ti_3_AlC_2_ powders were purchased from Yiyi Technology Co., Ltd. (Changchun, China). Hydrochloric acid (12 M) was purchased from Fengchuan Chemical Reagent Co., Ltd. (Tianjin, China). LiF (99.99%) was purchased from RHAWN Chemical Technologies Co., Ltd. (Shanghai, China). Bisphenol-A diglycidyl ethers (DGEBA, E51) were purchased from Sinopec Baling Petrochemical Co., Ltd. (Yueyang, China). Methyl-5-norbornene-2,3-dicarboxylic anhydride (MNA, 95%, mixture of isomers) and 2-Ethyl-4-methylimidazole (2,4-EMI, 96%) were purchased from Aladdin Biochemical Technology Co., Ltd. (Shanghai, China). All the reagents required no further purification.

### 2.2. Preparation of MXene

LiF (1 g) and hydrochloric acid (20 mL, 9 M) were mixed and stirred for 30 min. Ti_3_AlC_2_ (1 g) was added to the reaction system and stirred for 24 h at 40 °C to obtain a suspension containing m-MXenes. The suspension was centrifuged (3500 rpm, 5 min), and the retained precipitate (m-MXene) was washed with deionized water. The process of centrifugation and washing was repeated several times until the pH of the supernatant was greater than 6. The precipitates were vacuum dried for 12 h to obtain m-MXene powders. To prepare f-MXene, m-MXene powders were dispersed in anhydrous ethanol. The ethanol suspension of m-MXene was sonicated for 1 h and then centrifuged (3500 rpm) for 5 min. The upper liquid layer obtained by centrifugation was the suspension of f-MXene. The centrifuged precipitate was dispersed into ethanol, and the sonication and centrifugation procedures were repeated to obtain more f-MXene suspensions. The f-MXene ethanol suspension was filtered using a tetrafluoroethylene filter membrane and washed with deionized water. The filter membrane loaded with f-MXene was sonicated in deionized water to obtain an aqueous suspension of f-MXene. The suspension was freeze dried for 24 h to obtain f-MXene powder. The freeze-drying procedure has the advantage of preventing the re-stacking of the nanosheets to maintain the morphology of few layers.

### 2.3. Preparation of MXene/EP Composites

Firstly, an MXene/MNA colloidal suspension was prepared as a curing agent. A certain amount of m/f-MXene was mixed with MNA, with the mass fraction of the nanosheets corresponding to 0.21% of the suspension. After mechanical stirring and sonication treatment, a uniform and stable colloidal suspension was obtained. These processes were carried out at room temperature. Then, DGEBA, an MXene/MNA suspension, and 2,4-EMI (as a promoter) were mixed in a mass ratio of 100:90:1. The mixture was thoroughly stirred and degassed under a vacuum environment to obtain an MXene/EP suspension with 0.1 wt.% MXenes. Finally, the MXene/EP suspension was slowly poured into a mold and heat-cured (120 °C for 2 h and 150 °C for 3 h) to obtain the required MXene/EP composite samples. Ultimately, three samples were prepared, including 0.1 wt.% m-MXene/EP, 0.1 wt.% f-MXene/EP, and pure EP without MXene.

### 2.4. Aging

The aging method employed was hydrothermal aging, and the experiment was conducted using a water bath. The samples to be aged were directly immersed in deionized water, and the water temperature was heated to 100 °C. Samples were removed at certain intervals for property testing. The maximum aging time was 400 h.

### 2.5. Characterization

#### 2.5.1. Tensile

The tensile strength was tested using a tensile testing machine (INSTRON 5565, Norwood, MA, USA), and according to ASTM D638 [35]. The specimen was of a dumbbell type with a thickness of 4 mm. The number of valid tests for each state of the sample was five.

#### 2.5.2. Microstructure

Morphological examinations were carried out using a scanning electron microscope (SEM, Phenom XL, Rotterdam, The Netherlands) and transmission electron microscopy (TEM, Hitachi H7650, Tokyo, Japan).

#### 2.5.3. Water Absorption

The water absorption *W* was calculated from the measured weights of the dried *M*_dry_ and wet samples *M*_wet_ through Equation (1) under normal atmospheric pressure. The temperature of the water was set to 25 °C and 100 °C. The number of valid tests for each state of the sample was five.

(1) $$W=\frac{M_{\mathrm{wet}}-M_{\mathrm{dry}}}{M_{\mathrm{dry}}}\times 100\%$$

#### 2.5.4. Fourier Transform Infrared (FTIR) Spectroscopy

The functional groups present in the dispersed MXene, MNA, and MXene/MNA samples were characterized using a Fourier transform infrared (FTIR, Nicolet iS50, Waltham, MA, USA) Spectrometer. The wavenumber range was from 400 to 4000 cm^−1^. The MXene powder sample was prepared via grinding and tableting with KBr. Since the number of layers in the nanosheets does not affect the type of surface functional groups, s-MXene was used as a representative. The MNA sample was in its original form without any treatment. The MXene/MNA sample was formed into a wet film after the suspension was filtered through a polytetrafluoroethylene microporous membrane using vacuum filtration. The purpose of filtration was to remove excess MNA to ensure the surface of the MXene was fully exposed.

#### 2.5.5. Differential Scanning Calorimetry (DSC)

The endothermic or exothermic behavior during the curing of liquid resin was measured using DSC equipment. The temperature range was from 25 °C to 220 °C. Four heating rates were employed, which were 5, 10, 15, and 20 °C/min. The same equipment was also used to measure the *T*_g_ of the resin-cured product. The temperature program was as follows: heating from 25 °C to 220 °C, then cooling from 220 °C to 25 °C, and finally re-heating from 25 °C to 220 °C. The rate for each stage was 10 °C/min. The number of valid tests for each state of the sample was three.

## 3. Results and Discussion

### 3.1. Micromorphology

**Figure 2:**
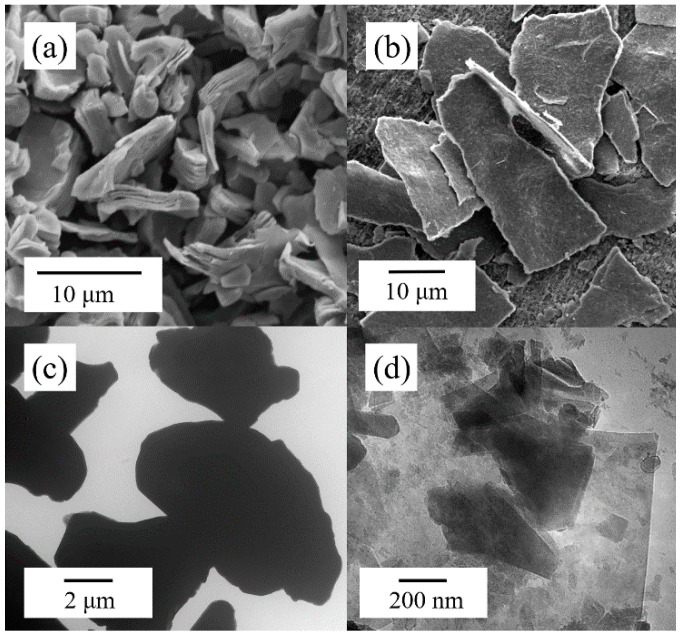
Microphotographs of MXene. (**a**) SEM image of m-MXene. (**b**) SEM image of f-MXene. (**c**) TEM image of m-MXene. (**d**) TEM image of f-MXene.

Figure 2 shows the SEM image and TEM image of MXene. After etching, the m-MXene showed an accordion-like stacked morphology (Figure 2a). After 1 h of sonication for delamination, the tightly stacked layer structure of the MXene sheets transformed into single- or few-layered sheet structures (Figure 2b). The f-MXene sheets in the TEM image are almost transparent, indicating thinness and a high degree of delamination (Figure 2d). Notably, Lu Liu et al. [26] reported the delamination procedure of m-MXene in methyl tetrahydrophthalic anhydride. In our work, the preparation process was delamination (in ethanol) and then dispersing (in MNA). We also tried stripping MXene in MNA. However, after the sonication process, the majority of the nanosheets in the m-MXene/MNA suspension remained in a multilayered morphology (Figure 2c). It is challenging to delaminate into f-MXene in MNA. This may be due to the significantly higher viscosity of MNA compared to methyl tetrahydrophthalic anhydride. Therefore, a preliminary delamination step was necessary to prepare the f-MXene/MNA suspension.

### 3.2. Functional Group Analysis

**Figure 3:**
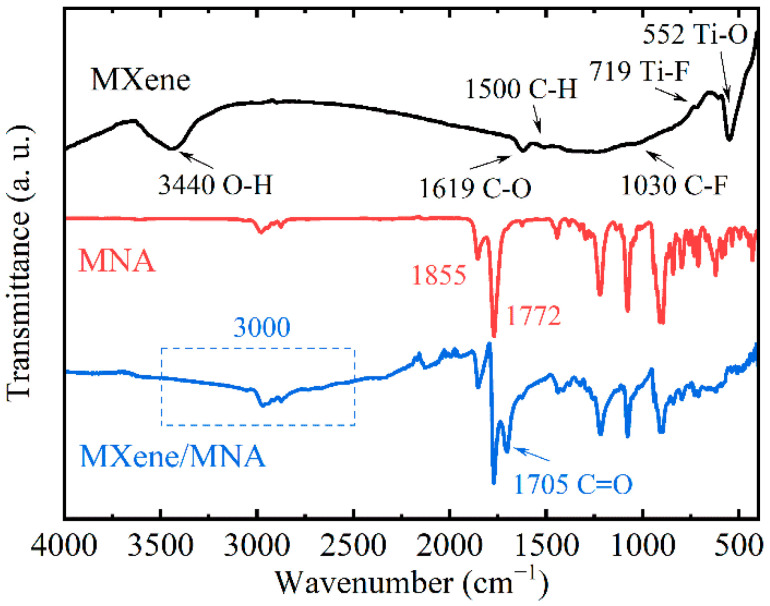
FTIR spectra of MXene, MNA, and MXene/MNA.

**Figure 4:**
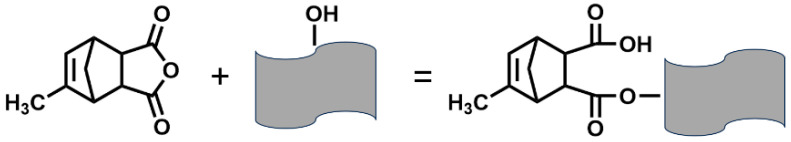
Chemical bonding formed between MNA and MXene.

FTIR spectroscopy was used to characterize the surface functional groups of MXene, and C-O (1619 cm^−1^), Ti-H (1510 cm^−1^), C-O (1244 cm^−1^), C-F (1030 cm^−1^), and Ti-O (552 cm^−1^) groups were observed on the surface of the MXene (Figure 3). Additionally, a broad peak near 3440 cm^−1^ confirmed the presence of hydroxyl groups. Similar FTIR spectra were observed by Tetiana Parker et al. [36], verifying the existence of various functional groups. Characteristic functional groups of MNA at 1845 cm^−1^ and 1770 cm^−1^ were identified as carbonyl groups [37]. MXene/MNA had a new peak at 1700 cm^−1^ and a clear broad peak around 3000 cm^−1^, which was identified as a carboxyl group [26]. Based on the changes in the FTIR spectra, it can be inferred that the anhydride ring of MNA can react with hydroxyl groups on the surface of MXene to form carboxylic acid groups and chemical bonds. The schematic diagram in Figure 4 shows the chemical bonding formed between MNA and MXene.

**Figure 5:**
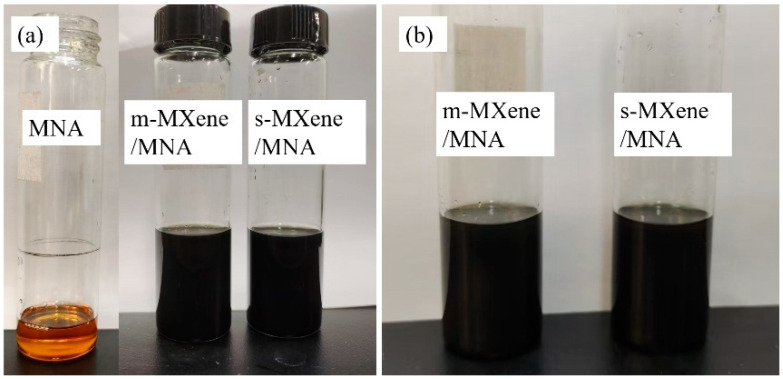
Stability of MXene/MNA suspensions. (**a**) Photograph of the suspension just prepared. (**b**) Photograph after 144 h of storage.

The chemical bonding between MNA and MXene offers two significant advantages. Firstly, it promotes the dispersion of MXene in MNA. Figure 5 presents a photograph of the MXene/MNA suspension stored in a glass bottle for 144 h. It can be observed that neither f-MXene nor m-MXene showed significant settling. The high dispersibility and stability of the suspension ensured the uniform distribution of MXene in the epoxy resin matrix. Secondly, the chemical bonding enhanced the interfacial strength between the nanosheets and the resin. In the MXene/EP system, a strong interface is formed between the nanosheets and the resin, rather than just a physical interaction.

### 3.3. Curing Kinetics

**Figure 6:**
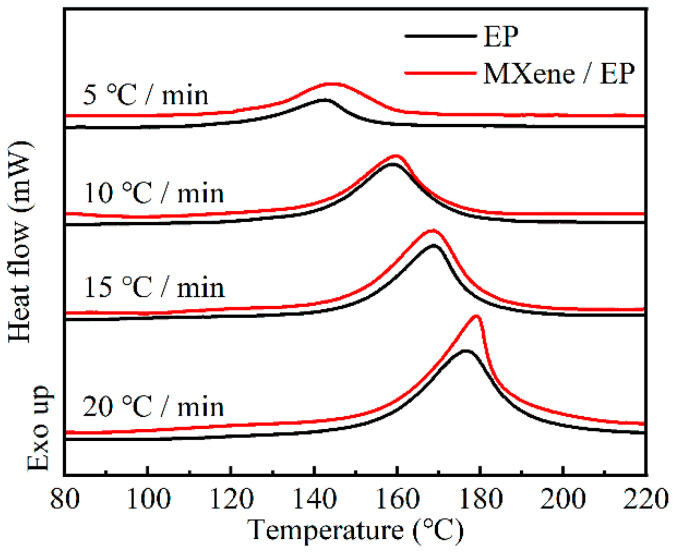
DSC curves for curing.

To investigate the influence of MXene on the curing process of epoxy resins, the exothermic and endothermic behaviors of the pure epoxy (EP) system and the MXene/EP system during curing were analyzed using non-isothermal DSC. Figure 6 displays the DSC curves of both systems at different heating rates (*q*), which exhibit similar behavior. Both systems display a single exothermic peak during the curing process. As the *q* value increases, the exothermic peaks of each system gradually shift to higher temperature regions, and the peak value also increases. This phenomenon can be attributed to the fact that at lower *q* values, the system approaches equilibrium, which prolongs the curing reaction time. This favors a slow exothermic curing reaction, which manifests as a wide and shallow peak. Increasing the reaction rate leads to a significant temperature gradient and heat release per unit time, resulting in a narrow and sharp exothermic peak [38]. Furthermore, the initial temperature (*T*_i_), peak temperature (*T*_p_), and final temperature (*T*_f_) of the curing reaction at different heating rates can be obtained from the DSC curves. By extrapolating the characteristic temperatures, the characteristic temperatures at a heating rate of 0 can be determined (Supplementary Information Figure S1). This information is valuable for guiding the design of curing processes. The results showed that the characteristic temperatures of the system shifted slightly towards higher temperatures after the addition of MXene. The characteristic temperatures of pure epoxy were 114.5 °C, 133.7 °C, and 144.2 °C (*T*_i_, *T*_p_, *T*_f_), respectively, while those of MXene/EP were 118.1 °C, 134.7 °C, and 153.3 °C (*T*_i_, *T*_p_, *T*_f_), respectively. Rui Cai et al. [28] obtained similar results in their study of MXene/phenolic epoxy composites with different amine curing agents. They attributed this to the increased energy barrier in the initial stage of the reaction and the participation of -OH groups on the MXene surface in the curing reaction [28,39,40]. Jing Zhang et al. [41] also observed an increase in the characteristic temperatures of the system in their study of graphene and carbon nanotube epoxy composites with different anhydride curing agents. They considered that the presence of nanofillers slowed down the curing process due to spatial steric hindrance effects. The apparent reaction activation energy (*E*_a_) is a crucial parameter for studying the curing behavior of epoxy systems. We used the Kissinger equation (Equation (2)) [42] to calculate the driving energy of the EP curing reaction.

(2) $$\ln(\frac{q}{T_{p}^{2}})=\ln(\frac{AR}{E_{a}})-\frac{E_{a}}{RT_{p}}$$

where *T*_p_ is the exothermic peak temperature, *q* is the heating rate, *A* is a factor, and *R* is the gas constant (8.314 J/(mol⋅K)).

The details of the calculation are provided in Figure S2 and Table S1 in the Supplementary Information. The calculated results showed that the activation energy of pure EP was 56.4 kJ/mol, while the activation energy of MXene/EP was 56.6 kJ/mol. The difference was negligible, indicating that the addition of 0.1 wt.% MXene did not show a significant promotion or inhibition effect on the curing of the epoxy resin in this study.

### 3.4. Glass Transition Temperature

**Figure 7:**
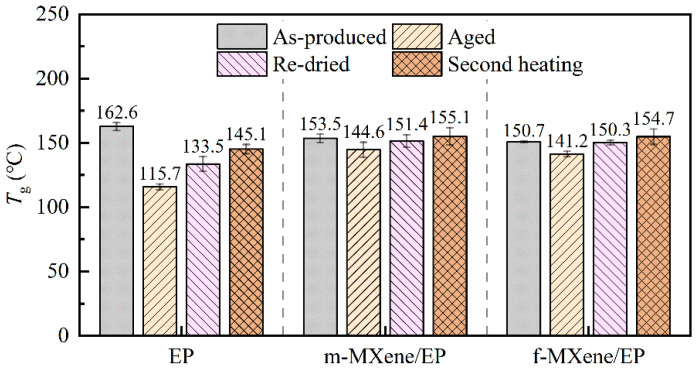
Glass transition temperatures of pure EP, MXene/EP (as-produced, aging, re-dried, and second heating).

The *T*_g_ values of the samples were obtained from DSC curves (Supplementary Information Figure S3) as shown in Figure 7. Compared to pure epoxy, the initial *T*_g_ values of all the MXene/epoxy composites decreased slightly, which is similar to the report by Lu Liu et al. [26]. The error bars for MXene/EP have no overlap with those for pure EP and are therefore considered to be significantly different. The influence of nanofillers on the *T*_g_ of epoxy resin may involve various mechanisms [43]. Some studies have reported an increase in *T*_g_ for carbon nanotube- or graphene-reinforced epoxy resins [17,44]. Some other reports have observed a decrease in *T*_g_ for composites reinforced via the covalent incorporation of nanofillers [26,43,45,46]. This decrease in *T*_g_ may be attributed to the covalently incorporated nanofillers reducing the crosslink density of the epoxy network. One explanation suggests that due to the broad relaxation temperature range in nanocomposites, MXene restricts the mobility of adjacent epoxy chains through strong interfacial bonding. Consequently, different relaxation behaviors occur for chains far away from the interface [26,45]. Additionally, the nature of the matrix also plays a crucial role, with the effects of nanofillers differing between highly crosslinked and low crosslinked matrices. In highly crosslinked thermosetting resins, nanofillers may disrupt the crosslinking network of the system, reducing the effective crosslink density and leading to a decrease in *T*_g_ [43]. In our work, MXene and the EP system are covalently bonded, and the pure resin matrix itself has a high crosslink density (*T*_g_ reaching 162.6 °C). Therefore, these mechanisms may have contributed to the decrease in *T*_g_ for MXene-reinforced epoxy resin composites. Moreover, the *T*_g_ average value of the s-MXene/EP system is slightly lower than that of the m-MXene/EP system. Since the s-MXene has a larger specific surface area, meaning that it has more surface groups per unit mass at the same weight fraction, the lower *T*_g_ is reasonable. It should be noted that the statistical difference between the two MXene/EP data is not significant (Supplementary Information Table S3). More experiments are needed to confirm the effect of the number of MXene layers on the initial *T*_g_.

High temperatures and water can cause the plasticization and hydrolysis of the resin matrix, thereby increasing the molecular mobility and reducing the *T*_g_ of the resin [13,16,44]. After 400 h of exposure to a hydrothermal environment, all three systems showed a decrease in *T*_g_. For the pure EP system, the *T*_g_ of the aged samples was significantly lower than that of the as-produced samples, dropping from 162.6 °C to 115.7 °C. After re-drying the aged samples, the *T*_g_ recovered to 133.5 °C, a significant recovery attributed to the reversible effect of moisture-induced plasticization. Similar phenomena were reported by Starkova et al. [13] in their study on graphene and carbon nanotubes. The *T*_g_ of the aged samples decreased drastically due to water plasticization and an increase in the free volume content. By removing the water that caused plasticization through re-drying, the glass transition temperature shifted to a higher value. However, the recovery of the *T*_g_ in the re-dried samples was limited and still lower than that of the as-produced samples, indicating the irreversible hydrolysis effects. We conducted further tests on the re-dried samples. We subjected the aged samples, which had undergone a heating program (25–220 °C), to a second test after cooling. This “heating-annealing” process is typically used to eliminate the thermal history of the material. The second heating test resulted in a higher *T*_g_ value than that of the dried samples, suggesting that the crosslink density of the aged samples could be further restored after exposure to high temperatures of 220 °C. However, the *T*_g_ value of the second heating test for the pure resin system was still lower than that of the as-produced sample. It further demonstrated the irreversible structural damage caused by hydrothermal aging to the resin.

The *T*_g_ values of both MXene/EP systems decreased much less than that of the pure EP system after hydrothermal aging, from 153.5 °C to 144.6 °C (m-MXene) and from 150.7 °C to 141.2 °C (s-MXene), respectively. A similar phenomenon was reported for modified graphene oxide by Starkova et al. [14]. The limiting effect of MXene on Tg deterioration was more pronounced in our experiments than with their study. This may be related to the material and aging conditions. Moreover, after re-drying, the *T*_g_ values of the samples completely recovered to their pre-aging levels. The statistical analysis of the data from the as-produced samples and the data from the re-dried samples showed that the differences were not significant (Supplementary Information Table S4). This indicates that the addition of MXene effectively limited the hydrolysis of the resin system. Furthermore, we conducted a second heating test on the MXene/EP systems. Interestingly, the *T*_g_ values of both MXene systems increased further, reaching 155.1 °C and 154.7 °C, respectively. These values are higher than those of the as-produced MXene/EP systems. As discussed at the beginning of this section, the addition of MXene led to a decrease in the *T*_g_ of the resin system. The results of the second heating test may be due to the high temperature affecting the interface structure between MXene and EP, resulting in a recovery of the crosslink density of the system. More experiments are needed to confirm the effect of high temperature on MXene/EP.

### 3.5. Water Absorption

**Figure 8:**
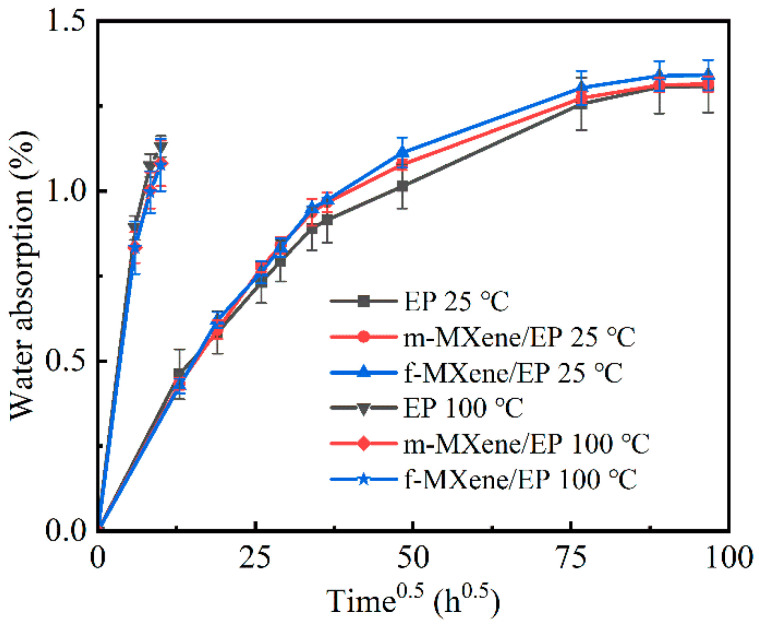
Water absorption of pure EP and MXene/EP (room temperature and 100 °C).

Figure 8 shows the mass gain during the 9360 h exposure in a water bath at room temperature. It can be clearly seen from the figure that the adsorption curves of the three samples are highly similar. The adsorption curves are linear in the early stage of water absorption. The samples start to approach saturation after 1320 h, and the water content of all three material systems stabilizes at around 1.3%. This water absorption behavior is consistent with the Fick model [9]. Given that the length (50 mm) and width (50 mm) of the samples are significantly larger than the thickness (4 mm), the water diffusion can be considered a one-dimensional diffusion problem. The water diffusion coefficient *D* can be estimated from the initial linear portion of the mass gain *M*(*t*) versus the square root of time *t*^0.5^ (Equation (3)).

(3) $$D=\frac{\pi }{16M_{\infty }}\left(\frac{M(t)}{\sqrt{t}}h\right)$$

where *D* is the diffusion coefficient [mm^2^/h], *M_∞_* is the equilibrium absorption rate [%], *t* is the aging time [h], *M*(*t*) is the water absorption rate at time *t*, and *h* is the thickness of the sample [mm]. The diffusion coefficients of EP, m-MXene/EP, and s-MXene/EP are 0.0152 mm^2^/h, 0.0158 mm^2^/h, and 0.0157 mm^2^/h, respectively. The differences in water diffusion among the three systems are minimal. As shown in Figure 7, the error bars of the samples overlap significantly at each time point. Subsequently, we tested the mass gain during the exposure to a hot water bath for 100 h. Compared to room temperature, the water absorption rate of all three samples increased. However, as in the room temperature experiment, the differences among the three samples remained minimal. Since the three samples did not show differences in the initial linear phase, longer water-absorption experiments at 100 °C were not conducted. Despite the differences in thickness, the water absorption behavior of the nanocomposites filled with s-MXene and m-MXene was similar. These results seem to differ from those reported for graphene or carbon nanotubes. Starkova et al. [13] reported the study of epoxy resin filled with thermally reduced graphene oxide and multi-walled carbon nanotubes. They found that the addition of a small amount of carbon nanofillers greatly reduced the amount of water absorbed, but the diffusion rate was not significantly affected by the nanofillers. The difference in water absorption between the three samples might be related to the following factors. Firstly, based on the general view from previous research, nanofillers act as barriers to hinder water diffusion, resulting in a more tortuous path for water molecules to enter the nanocomposites. Meanwhile, in the case of strong bonding, nanofillers restrict the movement of polymer chains around the particles, thereby retarding the relaxation of polymer chains. This effect may be more pronounced in long-time high-temperature experiments. These factors play a positive role in limiting water absorption. However, as analyzed in Section 3.4, due to the high cross-linking density of the pure resin formulation itself used in our study, the addition of MXene resulted in a decrease in the cross-linking density. The decrease in the cross-linking density led to an increase in the free volume, which provided favorable conditions for the penetration of water molecules. Additionally, MXene surfaces have a large number of hydrophilic groups, which is also a factor that contributed to the difference in water absorption between MXene/EP and reduced graphene oxide/EP. These favorable and unfavorable factors worked together to ultimately lead to the close similarity between EP and MXene/EP in terms of water absorption. Carey MS. et al. [25] investigated the water transport properties of MXene/EP composites in a vapor environment and found that the water absorption rate of MXene/EP was significantly lower compared to that of pure EP. However, unlike our work, they exchanged the lithium ions between the MXene multilayers with di (hydrogenated tallow) benzyl methyl ammonium chloride or 12-aminolauric acid to change the interlayer space properties from hydrophilic to hydrophobic. Moreover, the MXene loading in their study was 5 wt.%, significantly higher than our loading of 0.1 wt.%. These differences might be the key reasons for the different experimental results. The study of the water absorption mechanism of MXene/EP composites is still in its early stages. Further elucidating the role of the various factors that affect the water absorption behavior in epoxy resin composites, especially quantitative analysis, will be the direction of future work.

### 3.6. Tensile Properties’ Evolution

**Figure 9:**
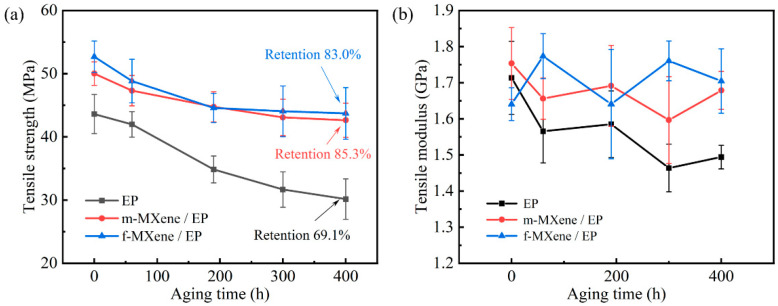
Evolution of tensile properties under hydrothermal conditions (100 °C water immersion). (**a**) Tensile strength. (**b**) Tensile modulus.

Figure 9a presents the evolution of tensile strength for pure EP, s-MXene/EP, and m-MXene/EP under hydrothermal conditions, with all the samples dried prior to testing. Notably, the addition of MXene resulted in initial strength increases of 14.7% (m-MXene) and 20.9% (s-MXene) compared to the pure EP sample. A similar strength enhancement has been observed in the other studies of MXene or nanofillers [47,48,49]. The microscopic examination of the tensile fracture surfaces of the samples also provided further evidence of this mechanism (Supplementary Information Figure S4). The quantitative analysis of surface roughness, crack propagation, or other relevant features will be the focus of future work. With the progression of hydrothermal aging, the tensile strength of all samples gradually decreased. After 400 h of hydrothermal aging, the tensile strength retention for the pure EP sample was 69.1%, while the retention rates for the MXene/EP samples were 85.3% (m-MXene) and 83.0% (s-MXene). The analysis of variance showed that the effects of aging time and MXene addition on the strength and retention of the resin were significant (Supplementary Information S3, the data was provided in Table S5). A similar enhancement has been observed in the other studies of nanofillers [50]. The increase in strength retention shows that MXene enhances the resistance of the resin system to hydrothermal aging. This phenomenon can be explained from two aspects. Firstly, MXene reduced the plasticization and hydrolysis of the resin matrix. MXene limited the water transport and molecular chain migration. Secondly, the stress transfer ability of the nanofiller partially mitigated the adverse effects of hydrothermal aging on the composite materials [51]. Due to the complexation of the failure mechanism in the composites, the degradation of the resin matrix had a reduced impact on the overall strength of the composites. Comparisons between the mechanical property evolutions of s-MXene/EP and m-MXene/EP revealed an overall consistency. Although there are differences in the mean values, there are no significant differences in the mechanical properties between the s-MXene and m-MXene systems considering the high overlap of the error bars. We also provide information about the tensile modulus as shown in Figure 9b. As the aging progresses, the tensile modulus of the pure EP shows an overall decreasing trend. The retention of tensile modulus after 400 h is 87.2%, which is small relative to the change in tensile strength. However, the tensile modulus of MXene/EP fluctuated around 1.7 GPa throughout the aging period. Since the magnitude of the fluctuation was within the individual error of the specimen, it was judged that there was no significant deterioration of the tensile modulus of MXene/EP during the 400 h aging period.

## 4. Conclusions

In this work, we prepared low-loading (0.1%) Mxene/EP composites cured with an anhydride and discussed the preparation process and hydrothermal resistance of the composites.

(1) Stable m-MXene/MNA suspensions and f-MXene/MNA suspensions could be prepared by directly dispersing or via first delamination and then dispersing, respectively, using the curing agent, MNA, as a solvent. Based on the changes in the FTIR spectra, it can be inferred that the anhydride ring in MNA could react with the hydroxyl groups on the MXene surface to form carboxylic acid groups and undergo chemical bonding. The obtained suspensions exhibited high stability with no significant settlement observed within 144 h. Cure kinetics analysis showed that the addition of MXene shifted the characteristic temperatures of the system towards higher temperatures. The start temperature, peak temperature, and end temperature of the curing reaction increased by 3.6, 1.0, and 9.1 °C, respectively. However, the change in the activation energy of the curing reaction was minimal, indicating that trace MXene had no significant promoting or inhibiting effect on the curing of the epoxy resin system in this study.

(2) The addition of MXene reduced the initial cross-linking density of the epoxy resin and resulted in a decrease in the *T*_g_ value. The initial *T*_g_ of m-MXene/EP decreased from 162.6 °C for pure resin to 153.5 °C. Due to the higher number of surface functional groups on f-MXene, the initial *T*_g_ decreased to 150.7 °C. After hydrothermal aging, the *T*_g_ of pure EP decreased by 46.9 °C, and drying the samples did not completely restore the *T*_g_. The decrease in the *T*_g_ value after hydrothermal aging for the MXene/EP system was much smaller than that for the pure EP system. After hydrothermal aging, the *T*_g_ values of the MXene/EP system decreased by 8.9 °C (m-MXene) and 9.5 °C (f-MXene), respectively. Moreover, after drying, the *T*_g_ values of the samples completely recovered to their pre-aging levels. This indicated that the addition of MXene effectively limited the plasticization and hydrolysis of the resin system.

(3) Water-absorption experiments (25 °C and 100 °C) showed that the water-absorption behavior of the three systems was extremely similar. This phenomenon was likely the result of various positive and negative factors. A more in-depth clarification of the mechanisms by which MXene affects water-absorption behavior in epoxy resin composites will be the direction of future work.

(4) Tensile testing showed that the addition of MXene increased the initial strength of the resin system by 14.7% (m-MXene) and 20.9% (f-MXene). After 400 h of hydrothermal aging, the tensile strength retention rate for the pure EP sample was 69.1%, while the retention rates for the MXene/EP samples were 85.3% (m-MXene) and 83.0% (s-MXene). The increase in strength retention shows that MXene enhances the resistance of the resin system to hydrothermal aging.

In summary, MXene could form stable and strong interfacial composites with the anhdride-cured epoxy resin system. The addition of only 0.1% loading of MXene could effectively improve the hydrothermal resistance of the epoxy resin.

## Data Availability

The original contributions presented in this study are included in the article/Supplementary Material. Further inquiries can be directed to the corresponding author.

## Supplementary Materials

The following supporting information can be downloaded at: https://www.mdpi.com/article/10.3390/polym17091229/s1, Figure S1: Characteristic temperatures for curing; Table S1: Calculated information on curing activation energy; Figure S2: Fits for the calculation of the activation energy of curing; Figure S3: Typical DSC curves of EP, MXene/EP samples (as-produced, aged, re-dried and second heating); Table S3: Comparing the significant differences between A5 and A9; Figure S4: SEM images of tensile section; Table S4: Comparing the significant differences between A5 and A7; Table S5: Tensile strength values for each sample.
