# Supplementary material for: Low MXene Loading of Epoxy Composite with Enhanced Hydrothermal Resistance
Source: Polymers (Basel). 2025 Apr 30;17(9):1229. doi: 10.3390/polym17091229 (PMC12073684; doi:10.3390/polym17091229)
Supplement: Supplementary file 1 [file polymers-17-01229-s001.zip › polymers-3556981-supplementary.pdf]

**Supplementary Information**

**Addition of low loading MXene to enhance hydrothermal resistance of epoxy resin**

Mengke Jing[1], Shujie Zhang[1*], Sichang Zhang[2], Mingzhou Li[1], Fan Chen[2], Yuchen Ma[2], Bo Sun[2]

[1] School of Textile Science and Engineering, Tiangong University, Tianjin 300387, China;

2 Beijing Gas Huanneng Engineering & Technologies Co., Ltd, Beijing 100020, China

*S1. Curing kinetics*

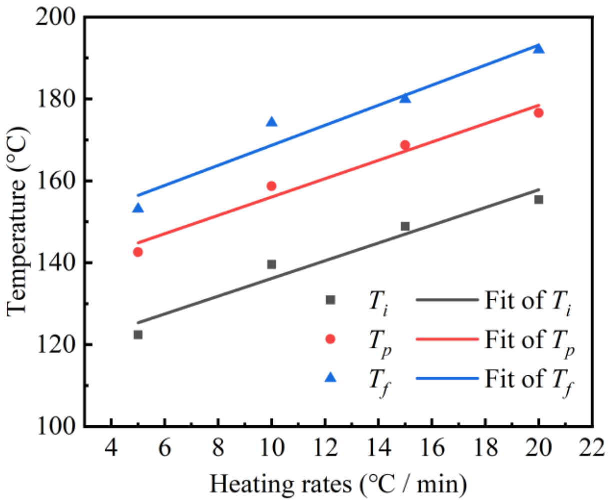


Figure S1 Characteristic temperatures for curing.

Table S1 Calculated information on curing activation energy.

| Samples | $q$ (K/min) | $T_p$ (K) | $1/T_p \times 10^3$ ($K^{-1}$) | ln ($q/T_p^2$) | $E_a$ (kJ/ mol) |
|---|---|---|---|---|---|
| EP | 5 | 415.75 | 2.40 | -10.45 | 56.4 |
| | 10 | 431.85 | 2.31 | -9.83 | |
| | 15 | 441.85 | 2.26 | -9.47 | |
| | 20 | 449.75 | 2.22 | -9.22 | |
| MXene/EP | 5 | 417.45 | 2.39 | -10.45 | 56.6 |
| | 10 | 432.85 | 2.31 | -9.83 | |
| | 15 | 441.75 | 2.26 | -9.47 | |
| | 20 | 452.15 | 2.21 | -9.23 | |

* Corresponding author. E-mail address: zhangshujie@tiangong.edu.cn(S. Zhang)

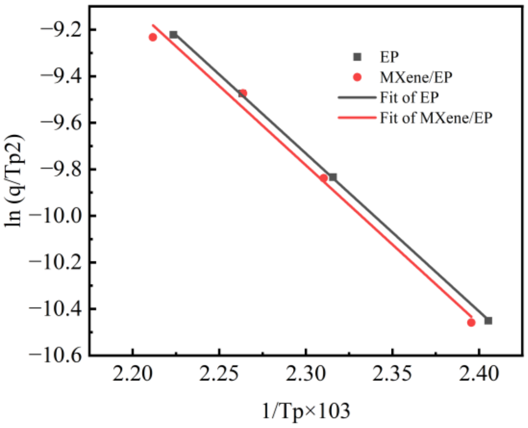


Figure S2 Fits for the calculation of the activation energy of curing.

*S2. Glass transition temperature*

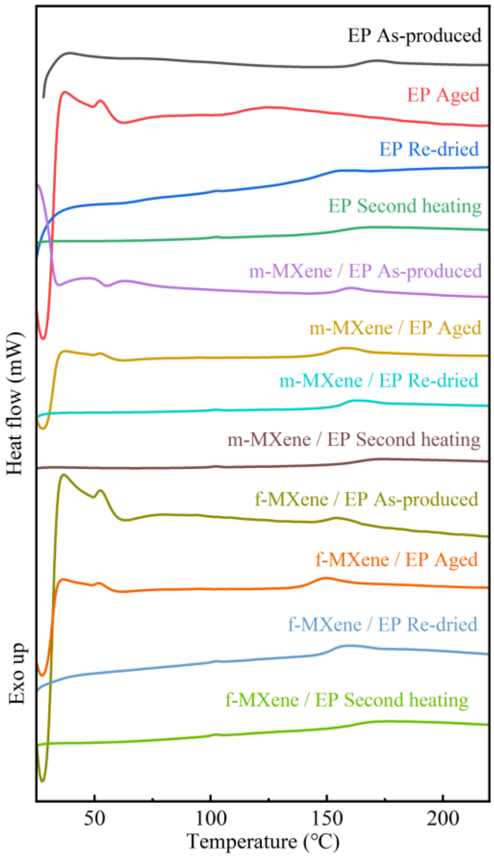


Figure S3 Typical DSC curves of EP, MXene/EP samples (as-produced, aged, re-dried and second heating).

Table S2 $T_g$ values for each sample

| Sample Type | Sample condition | Data | | | Data group number |
|---|---|---|---|---|---|
| EP | as-produced | 165.5 | 159.4 | 162.8 | A1 |
| | aged | 114.2 | 118.3 | 114.7 | A2 |
| | re-dried | 140.1 | 129.2 | 131.3 | A3 |
| | second heating | 141.0 | 146.3 | 147.9 | A4 |
| m-MXene/EP | as-produced | 157.0 | 151.3 | 152.1 | A5 |
| | aged | 151.2 | 142.8 | 139.9 | A6 |
| | re-dried | 157.1 | 148.1 | 149.2 | A7 |
| | second heating | 162.9 | 151.2 | 151.3 | A8 |
| s-MXene/EP | as-produced | 151.1 | 150.9 | 149.7 | A9 |
| | aged | 143.5 | 139.3 | 141.0 | A10 |
| | re-dried | 152.4 | 149.0 | 149.6 | A11 |
| | second heating | 161.5 | 152.5 | 150.1 | A12 |

Table S3 Comparing the significant differences between A5 and A9

| Methods of analysis | Process | | conclusion |
|---|---|---|---|
| Independent samples t-test | average value<br>153.47 150.57 | sample variance<br>9.52 0.57 | insignificant |
| | F-test<br>F=16.61 | critical value (two-tailed, $\alpha$=0.05)<br>$F_{0.025}(2,2)$ =19.00 | |
| | Pooled Variance<br>5.05 | t-value<br>1.58 | |
| | degrees of freedom<br>4 | critical value (two-tailed, $\alpha$=0.05)<br>$t_{0.975}$ (4) =2.776>1.58 | |

Table S4 Comparing the significant differences between A5 and A7

| Methods of analysis | Process | | conclusion |
|---|---|---|---|
| Independent samples t-test | average value<br>153.47 151.47 | sample variance<br>9.52 24.10 | insignificant |
| | F-test<br>F=2.53 | critical value (two-tailed, $\alpha$=0.05)<br>F 0.025(2,2) =19.00 | |
| | Pooled Variance<br>16.81 | t-value<br>0.598 | |
| | degrees of freedom<br>4 | critical value (two-tailed, $\alpha$=0.05)<br>t0.975 (4) =2.776>0.598 | |

*S3 Tensile properties evolution*

Figure S4 shows that the pure epoxy resin has a typical brittle fracture with a smooth surface. The samples of MXene/EP have rougher fracture surfaces than the pure epoxy resin samples, which suggests higher toughness of the nanocomposites. This phenomenon was judged to be the result of stress transfer between the matrix system and the nanofillers. MXene improves the fracture toughness of epoxy nanocomposites through energy dissipation, especially at low nanofiller contents.

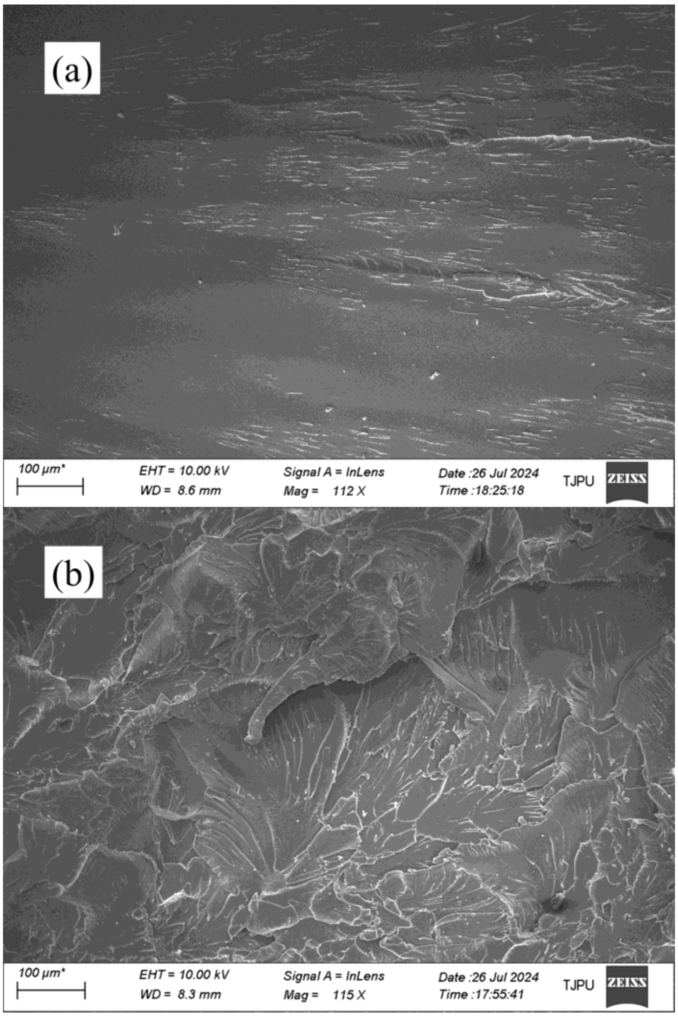


Figure S4 SEM images of tensile section.

Table S5 Tensile strength values for each sample

| | 0h (B1) | 60h (B2) | 190h (B3) | 300h (B4) | 400h (B5) |
|---|---|---|---|---|---|
| | 48.46 | 40.95 | 35.48 | 33.41 | 26.47 |
| EP | 41.82 | 40.34 | 32.019 | 28.32 | 33.58 |
| (A1) | 40.51 | 45.02 | 37.71 | 29.71 | 32.06 |
| | 42.63 | 40.51 | 33.66 | 35.31 | 31.59 |
| | 44.65 | 43.07 | 35.34 | 31.56 | 27.05 |
| | 48.42 | 44.87 | 41.83 | 45.31 | 45.48 |
| m-MXene | 51.50 | 49.05 | 42.57 | 44.69 | 40.61 |
| (A2) | 52.49 | 45.88 | 47.18 | 38.21 | 39.07 |
| | 49.13 | 50.59 | 46.14 | 44.47 | 44.42 |

| | | | | | |
|---|---|---|---|---|---|
| | 48.51 | 46.17 | 46.04 | 42.69 | 43.61 |
| s-MXene (A3) | 49.52 | 45.19 | 46.96 | 45.26 | 46.47 |
| | 55.44 | 54.39 | 47.16 | 38.87 | 39.49 |
| | 53.57 | 47.40 | 42.32 | 48.77 | 48.26 |
| | 50.64 | 49.29 | 43.54 | 44.83 | 47.18 |
| | 54.21 | 47.89 | 42.92 | 39.50 | 41.81 |

A hypothesis test using two-way ANOVA with equal replication was employed to examine whether the addition of MXene and temperature had statistically significant effects on tensile strength. Here, the addition of MXene was defined as **Factor A** with three levels (r=3), and temperature was defined as **Factor B** with five levels (s=5). Each combination of MXene addition and temperature was tested five times (c=5). Each tensile strength measurement is denoted as $X_{ijk}$, where:

- i=1,2,3 corresponds to the levels of Factor A (MXene addition),
- j=1,2,3,4,5 corresponds to the levels of Factor B (temperature),
- k=1,2,3,4,5 represents the five replicate measurements under each MXene-temperature combination.

For example, $X_{111}$ refers to the first tensile strength measurement under the combination of the first level of Factor A ($A_1$) and the first level of Factor B ($B_1$).

The sum of squares due to Factor A(SSA):

$$Q_A=\sum_{i=1}^{r}\sum_{j=1}^{s}\sum_{k=1}^{c}(\overline{X}i\cdot\cdot-\overline{X})^2=1604.03$$

The sum of squares due to Factor B(SSB):

$$Q_B=\sum_{i=1}^{r}\sum_{j=1}^{s}\sum_{k=1}^{c}(\overline{X}\cdot j\cdot-\overline{X})^2=1093.71$$

In statistical notation, this is commonly abbreviated as SSAB:

$$Q_I=\sum_{i=1}^{r}\sum_{j=1}^{s}\sum_{k=1}^{c}\left(\bar{X}_{ij\bullet}-\bar{X}_{i\bullet\bullet}-\bar{X}_{\bullet j\bullet}+\bar{X}\right)^2=25.97$$

The error sum of squares (SSE):

$$Q_E=\sum_{i=1}^{r}\sum_{j=1}^{s}\sum_{k=1}^{c}(Xijk-\overline{X}ij\cdot)^2=488.12$$

Total Sum of Squares(SST):

$Q_T=Q_A+Q_B+Q_I+Q_E=3211.83$

Given a significance level of α=0.05, the upper quantile $F_{0.05}$ is taken here (meaning there is a 95% confidence that significance can be determined), hence the analysis of variance table is available:

| source | Sum of squared deviations | Degrees of freedom (df) | Mean square deviation | F value ($S_A^2/S_E^2$) | critical value $F_{0.05}$ | significance |
|---|---|---|---|---|---|---|
| Factor | $Q_A$=1604.03 | 3-1=2 | $S_A^2=Q_A/2$ | 98.53 | $F_{0.05}(2,60)$ | significant |

| | | | | | | |
|---|---|---|---|---|---|---|
| A | | | =802.2 | | =3.15 | |
| Factor B | $Q_B$=1093.71 | 5-1=4 | $S_B^2= Q_B/4$ =273.43 | 33.59 | $F_{0.05}(4,60)$ =2.53 | significant |
| A×B | $Q_I$=25.97 | 2×4=8 | $S_I^2=Q_I/8$ =3.25 | 0.4 | $F_{0.05}(8,60)$ =2.1 | not significant |
| Error E | $Q_E$=488.12 | 3×5×（5-1）=60 | $S_E^2=Q_E/60$= 8.14 | | | |
| Total T | $Q_T$=3211.83 | 3×5×5-1=74 | | | | |

The calculation results from the table data show that when the addition of MXene changes, the F value is greater than $F_{0.05}$, indicating that the addition of MXene has a significant effect on tensile strength; When the temperature changes and the F value is greater than $F_{0.05}$, it indicates that the effect of temperature on tensile strength is also significant.

| source | Sum of squared deviations | Degrees of freedom (df) | Mean square deviation | F value | critical value $F_{0.05}$ | significance |
|---|---|---|---|---|---|---|
| Factor A | 1604.03 | 2 | 802.2 | 98.53 | 3.15 | significant |
| Factor B | 1093.71 | 4 | 273.43 | 33.59 | 2.53 | significant |
| A×B | 25.97 | 8 | 3.25 | 0.4 | 2.1 | Not significant |
| Error E | 488.12 | 60 | 8.14 | | | |
| Total T | 3211.83 | 74 | | | | |

Additionally, a one-way ANOVA was employed to test whether there were significant differences in the tensile strength retention rate (after 400 hours of hydrothermal aging) across three levels of MXene addition ($A_1$, $A_2$, $A_3$). The retention rate was calculated as the ratio of the tensile strength at 400 hours to the average initial tensile strength, with values rounded to two decimal places for computational convenience. The addition of MXene has three levels A1 A2、A3, We conducted *ni* experiments at each level *i* with a tensile strength of *Xij*. The average tensile strength under the addition of three different MXenes is ui

Make assumptions H0: u1=u2=u3 H1:ui Not completely equal

Statistics $F=\frac{S_A^2}{S_E^2}$ ~F（r-1，n-r），Rejection region W>$F_{0.05}$(r-1,n-r)

r=3,ni=5,n=15, So there is：

| MXene addition | $Xij$ | | | | | $\overline{X}i$ | $\sum_{j=1}^{ni}(Xij-\overline{X}i)^2$ | $ni(\overline{X}i-\overline{X})^2$ |
|---|---|---|---|---|---|---|---|---|
| A1 | 0.61 | 0.77 | 0.74 | 0.72 | 0.62 | 0.692 | 0.021 | 0.055 |
| A2 | 0.91 | 0.81 | 0.78 | 0.89 | 0.87 | 0.852 | 0.012 | 0.015 |
| A3 | 0.88 | 0.75 | 0.92 | 0.90 | 0.79 | 0.848 | 0.089 | 0.013 |

| | $Q_T$=0.205 | $\overline{X}$ = 0.797 | $Q_E$=0.122 | $Q_A$=0.083 |
|---|---|---|---|---|

Sum of squared deviations between groups：

$$Q_A=\sum_{i=1}^{r}\sum_{j=1}^{ni}(\overline{X}i-\overline{X})^2=\sum_{i=1}^{r}ni(\overline{X}i-\overline{X})^2=0.083$$

Mean square deviation $S_A^{\ 2}$ =0.042

Sum of squared deviations within the group：

$$Q_E=\sum_{i=1}^{r}\sum_{j=1}^{ni}(Xij-\overline{X}i)^2=0.122$$

Mean square deviation $S_E^{\ 2}$ =0.01

total sum of squares：

$$Q_T=\sum_{i=1}^{r}\sum_{j=1}^{ni}(Xij-\overline{X})^2=Q_A+Q_E=0.205$$

$$F=\frac{S_A^{\ 2}}{S_E^{\ 2}}=4.2$$

Column analysis of variance table：

| source | Sum of squared deviations | Degrees of freedom (df) | Mean square deviation | F value | critical value $F_{0.05}$ | significance |
|---|---|---|---|---|---|---|
| Factor A | 0.083 | 3-1=2 | 0.042 | 4.2 | $F_{0.05}(2, 12)$ =3.89 | significant |
| Error E | 0.122 | 15-3=12 | 0.01 | | | |
| Total T | 0.205 | 15-1=14 | | | | |

From the calculated results presented in the table data, it can be observed that the F-value exceeds the critical F-value at the 0.05 significance level ($F>F_{0.05}$) when varying MXene addition levels. This indicates that the addition of MXene has a statistically significant effect on the tensile strength retention rate.
